# Influence of Aortic Deviation on Abdominal Aorta Bifurcation Level Relative to Vertebral Position

**DOI:** 10.7759/cureus.73678

**Published:** 2024-11-14

**Authors:** Jyoti Kiran, Navbir Pasricha, Rajan Bhatnagar, Shamrendra Narayan, Anamika Gaharwar, Eti Sthapak

**Affiliations:** 1 Anatomy, Dr. Ram Manohar Lohia Institute of Medical Sciences, Lucknow, IND; 2 Radiology, Dr. Ram Manohar Lohia Institute of Medical Sciences, Lucknow, IND

**Keywords:** abdominal aortic bifurcation, abdominal aortic deviation, abdominopelvic ct scan, aortic variations, vertebral levels

## Abstract

Introduction

Research on abdominal aortic deviation (AAD) and its impact on the vertebral level of abdominal aortic bifurcation (AAB) has been limited. We aimed to determine the level of AAB with respect to vertebral levels and assess the proportion of AAD and its impact on AAB.

Materials and methods

This single-arm, cross-sectional, retrospective study involved contrast-enhanced computed tomography (CECT) scans of the abdomen and pelvis of 208 subjects aged 18 years or older. AAD and AAB in terms of vertebral levels were noted using the digital imaging and communication in medicine (DICOM) viewing software RadiAnt (Medixant, Poznań, Poland).

Results

The rate of AAD was found to be 44 out of 208 (21.2%), 16 out of 98 (16.3%) in men and 28 out of 110 (25.45%) in women (p=0.10). The AAD rates in the 31-40-, 51-60-, and 71-80-year age groups were 11 out of 37 (29.7%), 17 out of 61 (27.8%), and three out of seven (42.8%) with p=0.03, respectively. AAB was seen in the middle of the L4 vertebrae in 65 cases (31.2%), followed by 49 cases (23.6%) in the L4 lower vertebrae. Twenty-three (20.9%) women had AAB above the L4, compared to 13 (13.2%) men (p=0.34). AAB was located below the L4 vertebral level in eight out of 44 (18.18%) subjects with AAD versus 41 out of 164 (25%) subjects without AAD (p=0.55).

Conclusion

AAD was observed in approximately one-fifth of the subjects in our study cohort, with a higher proportion among women and in elderly age groups. AAB was most commonly observed in the middle of the L4 vertebrae and AAD did not impact AAB levels.

## Introduction

The abdominal aorta (AA) is the most important artery in the abdomen and is a continuation of the thoracic aorta after passing the diaphragm at the level of the 12th thoracic vertebra. Embryologically, the development of the aorta begins in the third gestational week with the dorsal migration of two cell lines from the endocardial mesenchyme. These cells grow along the neural groove to eventually fuse into a single aorta [1].

Abdominal aortic bifurcation (AAB) usually lies at the mid-level of the L4 lumbar vertebral body and branches into the left and right common iliac artery, which then further bifurcates into the external and internal iliac arteries. However, AAB may have several variations with respect to the vertebral level. Various studies have suggested that the typical AAB at the level of the L4 vertebral body is found in only 60-80% of cases [2-6]. AAB has been found to present significant variations, ranging from the upper half of the L3 vertebral body to the upper half of the L5 vertebral body [6]. Very rarely, AAB has been seen very low, at the level of the S2 vertebrae, or very high, at the level of the upper L2 body, both of which have been found to result in major clinical implications [7,8].

AAB varies with age. As individuals age, reduced intervertebral disc thickness results in the caudal shifting of AAB [9]. AAB also varies with sex. In their study with 100 participants, Khader et al. found that 70 (70%) of the men and 60 (60%) of the women had AAB at the L4 level [10]. Similarly, another study found that AAB was located higher in women compared with men [5].

Lumbosacral anomalies such as lumbarization and sacralization may lead to variations in AAB levels [4]. Other abnormalities, for example, the presence of cervical ribs, hypoplasia of the 12th rib, or transitional vertebra, may result in inaccurate vertebra counts and must be considered when the exact identification of the level of bifurcation is required [11]. Likewise, the presence of abdominal aortic deviation (AAD) may lead to variations in AAB levels [5]. The rate of AAD has been shown to be higher in women compared to men, and its presence may impact the level of AAB. Ekingen and Çetinçakmak reported no difference in AAB level in men versus women among individuals with AAD, while, in those without AAD, AAB was shown to be positioned higher in women compared to men [5].

Knowledge of variations in AAB level and the presence of AAD are crucial to avoid complications during different surgical procedures, such as anterior vertebral interbody fusion for spinal deformities like scoliosis, lordosis, and spinal trauma; laparoscopic approaches for discectomy; abdominal aortic aneurysm; aortic sclerosis; atherosclerosis; and abdominopelvic surgeries [12-14]. Knowledge of a patient's AAB is utilized in the two-dimensional planning of radiotherapy for carcinoma cervix (which is the second most common cancer among women in India) and for kissing stent reconstruction of aortoiliac disease [15,16].

While cadaveric research has offered data on anatomical variations, such studies have been restricted by the limited availability of cadavers, which results in small sample sizes, and are time-, resource-, and labor-intensive [12]. Advances in radiology have enabled the precise assessment of vascular anatomy [4,5], and contrast-enhanced computed tomography (CECT) is considered the gold standard, as it provides a clear picture of the vessels, including arteries and veins [17].

Studies on AAB exist but AAD and its impact on AAB levels among the Indian population have yet to be explored. As stated earlier, since AAD may influence the level of AAB and consequently impact surgical-anatomical decisions, it would be prudent to explore this association in our cohort of patients. The primary objective of our study was to determine the proportion of AA with and without AAD and compare AAD rates between men and women. The secondary objective was to determine the vertebral level of AAB and the effect of age and sex on AAB.

## Materials and methods

This was a single-arm, single-center, cross-sectional retrospective study undertaken in the Department of Anatomy and Radiology at Dr. Ram Manohar Lohia Institute of Medical Sciences, Lucknow, India, between September 2022 and March 2024. The study was approved by the institute's Institutional Ethics Committee (approval number: RC-433/RMLIMS/2022 dated 08/09/2022). CECT scans of the abdomen and pelvis of subjects aged 18 years and above were included. Patients with abdominal aortic and iliac pathology, such as abdominal aortic aneurysm and iliac thrombosis; spinal anomalies like kyphosis and scoliosis; major abdominal surgeries in the past; and poor-quality images were excluded.

Source of data and CT acquisition method

All CECT examinations were performed on a 64-slice multidetector CT scanner (Extended Brilliance Workspace, Version 6.4, Philips Medical Systems, Andover, Massachusetts, United States), which worked at 64×1 mm collimation with a minimum slice thickness of 0.625 mm. The machine operated at 120 kV and 320 mAs using standard departmental procedures. CECT scan images were retrieved retrospectively from the picture archiving and communication system (PACS) and then processed and analyzed with multiplanar reformation at the available workstation. Axial, coronal, and sagittal views were retrieved, along with maximum intensity projection and 3D reconstructed views. 

Aortic bifurcation level was noted in relation to the vertebral levels. To determine the location of AAB in terms of the level of the spine, four planes, including the vertebrae and intervertebral disc structure, were delineated: upper (above the level of the pedicle vertebra), middle (at the level of the pedicle vertebra), lower (below the level of the pedicle vertebra), and disc level (located at the level of the intervertebral disc).

In the images, the plane corresponding to AAB was accepted as the vertebral level of AAB. Images were loaded in the digital imaging and communication in medicine (DICOM) viewing software RadiAnt (Medixant, Poznań, Poland), and the vertebral levels of AAB were simultaneously cross-checked, loading sagittal, coronal, and axial images in the same viewing window. In the case of a zone overlap, the zone accommodating more than 50%, based on eyeballing, was assigned. 

Sample size calculation and statistics

The total sample size for the study was calculated to be 207 patients. The sample size was calculated using the primary objective of the study and based on the proportion of AAD, as reported by Ekingen and Çetinçakmak [5] and described as follows: $n=z^{2}p.q/d^{2}$. Here, z is 1.96 at a 95% confidence interval, p is the estimated proportion (here 16% or 0.16, i.e., 116/721 from the referenced study), q is $1-p=1-0.16=0.84$, and d is the desired precision (here taken as 5% or 0.05).

Patient demographics data (age, sex, and date of participation in the study) were acquired. AAD and vertebral levels of AAB were acquired and entered in a Microsoft Excel sheet (Microsoft Corporation, Redmond, Washington, United States). 

The obtained data was transferred and analyzed using IBM SPSS Statistics for Windows, Version 21.0 (Released 2012; IBM Corp., Armonk, New York, United States). Descriptive analysis was applied to summarize the data in terms of mean±standard deviation, median (range), frequency, etc. The independent Student's t-test (continuous variable) and chi-squared test (for categorical variable) were used to compare the groups according to gender and age. P-values with p<0.05 were accepted as statistically significant.

## Results

Two hundred and fifty-eight CECT images of the abdomen and pelvis were screened. CECT scans of 50 subjects were excluded (41 due to the poor quality of the images and nine due to insufficient slices of images for analysis). The final sample size included in the study was 208. The patient demographics are summarized in Table 1.

**Table 1:** Patient demographics

Patient attributes	Distribution (n=208)
Male-to-female ratio	98:110 (47% versus 53%)
Mean age of subjects	48.93 years
Median age of subjects (range)	50 (19-78 years)
Age	
18-30 years	22 (10.6%)
31-40 years	37 (17.8%)
41-50 years	55 (26.4%)
51-60 years	61 (29.3%)
61-70 years	26 (12.5%)
71-80 years	7 (3.4%)

AAD

The overall rate of AAD for both sexes combined was 44 out of 208 (21.2%). AAD was observed in 16 out of 98 men (16.32%) and 28 of 110 women (25.45%). A higher proportion of women had AAD compared to men; however, this result was not statistically significant (p=0.108).

**Figure 1:**
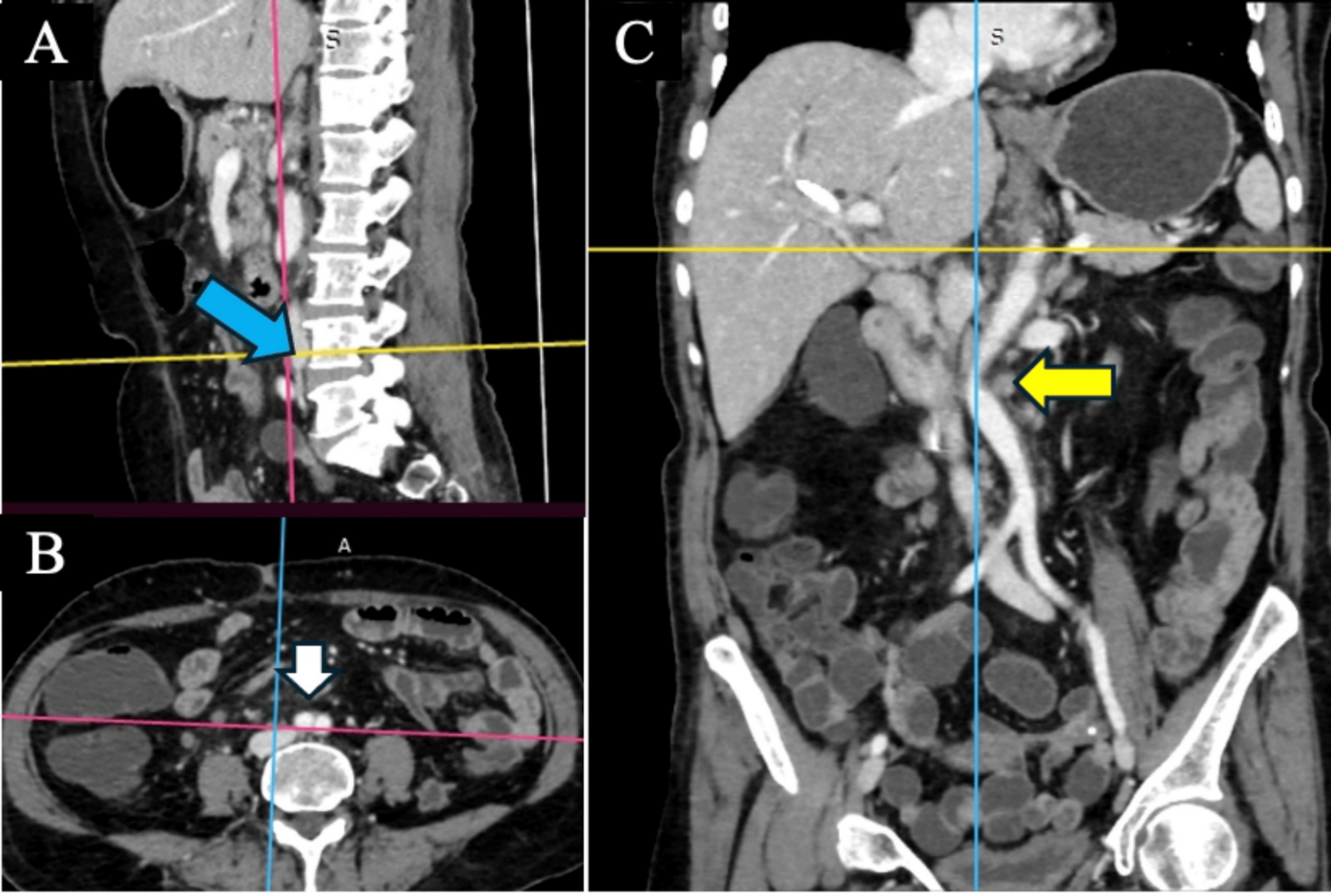
AAB in a case of AAD (A) AAB at the lower border of the L4 vertebrae (blue arrow). (B) AAB in the axial plane at the same level (white arrow). (C) AAD in the coronal plane (yellow arrow) AAB: abdominal aortic bifurcation; AAD: abdominal aortic deviation

**Figure 2:**
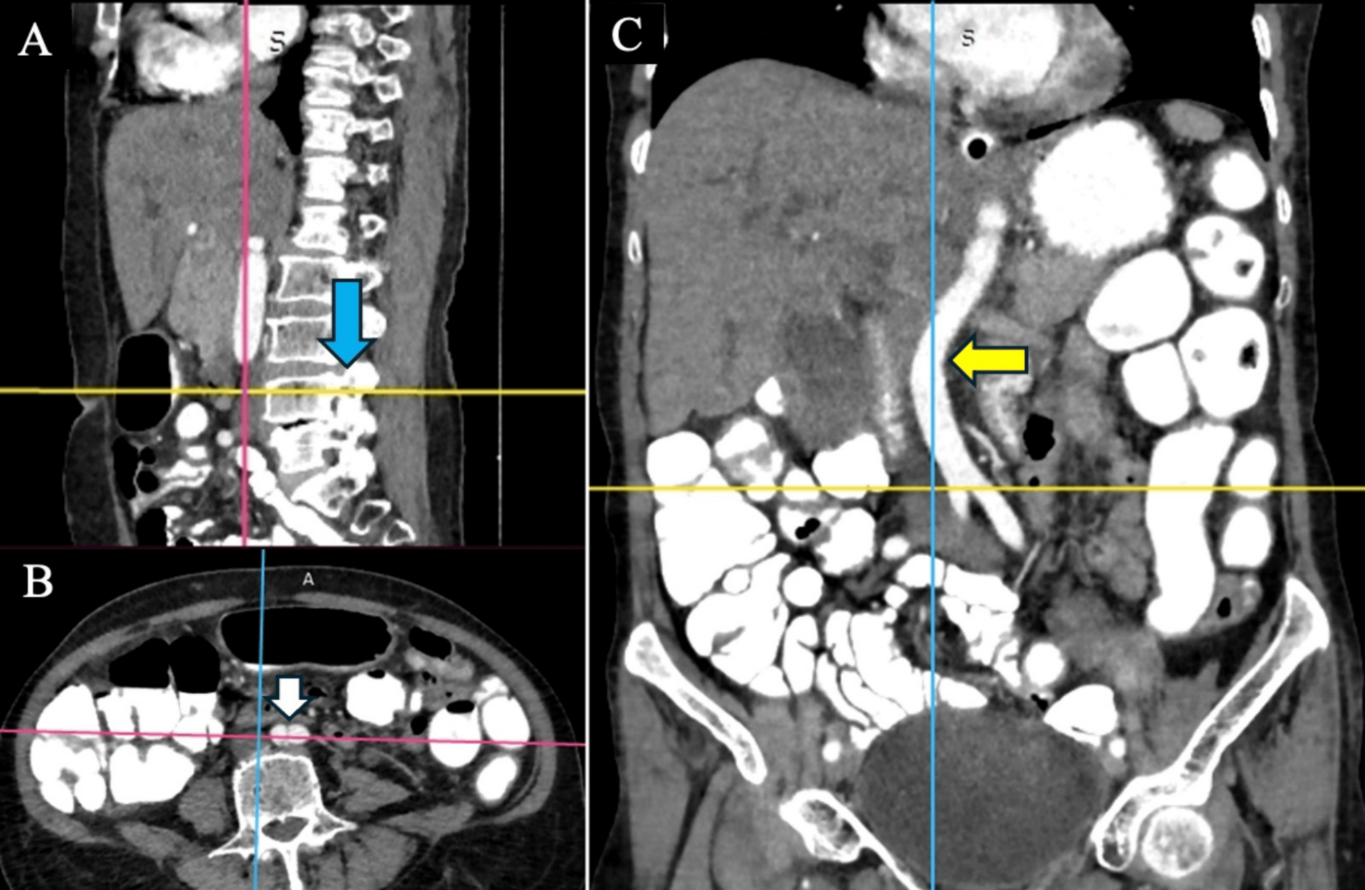
AAB at the upper L4 vertebrae in a subject with AAD (A) AAB at the upper L4 vertebrae in the sagittal plane (blue arrow). (B) AAB in the axial plane (white arrow). (C) AAD to the right of the midline in the coronal plane (yellow arrow) AAB: abdominal aortic bifurcation; AAD: abdominal aortic deviation

The maximum AAD rate was observed in the 71-80-year age group (3 subjects, 42.8%) followed by the 31-40-year age group (11, 29.7%) and 51-60-year age group (17, 27.8%), respectively. This difference in AAD rate was found to be statistically significant with a p-value of 0.0346 (Table 2). Figure 1 and Figure 2 represent AAD with AAB at the lower border and the upper border of the L4 vertebrae, respectively.

**Table 2:** Variation of AAD with respect to age AAD: abdominal aortic deviation

Age (years)	With AAD	Without AAD	Total	χ2	P-value
18-30	1 (4.5%)	21 (94.5%)	22	12.01	0.0346
31-40	11 (29.7%)	26 (70.3%)	37		
41-50	10 (18.2%)	45 (21.8%)	55		
51-60	17 (27.8%)	44 (72.2%)	61		
61-70	2 (7.7%)	24 (92.3%)	26		
71-80	3 (42.8%)	4 (57.2%)	7		
Total	44 (21.1%)	164 (78.9%)	208		

Levels of AAB

The most common position of AAB was the middle of the L4 vertebrae in 65 subjects (31.2%), followed by the L4 lower vertebrae in 49 (23.6%). In 123 subjects (59.1%), AAB was located at the level of the L4 vertebrae (either upper, middle, or lower). In 49 subjects (23.6%), AAB was located below the lower L4 vertebrae, and in 36 subjects (17.3%), AAB was located above the upper L4 vertebrae. The detailed frequency of the AAB rate as per vertebral levels is documented in Table 3. 

**Table 3:** Frequency of AAB as per the vertebral levels AAB: abdominal aortic bifurcation

Vertebral level of AAB	Number of subjects (percentage)
L2-L3 intervertebral disc	1 (0.5%)
L3 lower	10 (4.8%)
L3 middle	5 (2.4%)
L3 upper	1 (0.5%)
L3-L4 intervertebral disc	19 (9.1%)
L4 lower	49 (23.6%)
L4 middle	65 (31.2%)
L4 upper	9 (4.3%)
L4-L5 intervertebral disc	26 (12.5%)
L5 lower	5 (2.4%)
L5 middle	15 (7.2%)
L5 upper	1 (0.5%)
L5-S1 intervertebral disc	2 (1%)
Total	208

We noted a higher proportion of women, 23 of 110 (20.9%), as having AAB above the L4 vertebrae compared to 13 of 98 men (13.2%), although this result was statistically insignificant (p=0.346). Variations of AAB with respect to age are demonstrated in Table 4.

**Table 4:** Variation of AAB with respect to age AAB: abdominal aortic bifurcation

Age (years)	AAB above L4	AAB at L4	AAB below L4	Total	χ2	P-value
18-30	4	10	8	22 (10.6%)	9.52	0.482
31-40	8	24	5	37 (17.8%)		
41-50	12	28	15	55 (26.4%)		
51-60	6	40	15	61 (29.3%)		
61-70	5	17	4	26 (12.5%)		
71-80	1	4	2	7 (3.4%)		
Total	36 (17.3%)	123 (59.1%)	49 (23.6%)	208		

We could not find any statistical difference regarding the position of AAB in terms of age. Table 5 depicts AAB in patients with AAD and without AAD. A higher proportion of patients with AAD had AAB located below the L4 level, eight out of 44 (18.18%), compared with those without AAD, at 41 of 164 (25%), although this result was not statistically significant (p=0.55). 

**Table 5:** Variation of AAB with respect to AAD AAB: abdominal aortic bifurcation; AAD: abdominal aortic deviation

AAD	AAB above L4	AAB at L4	AAB below L4	Total	χ2	P-value
With AAD	7	29	8	44 (21.2%)	1.18	0.55
Without AAD	29	94	41	164 (78.8%)		
Total	36 (17.3%)	123 (59.1%)	49 (23.6%)	208		

**Figure 3:**
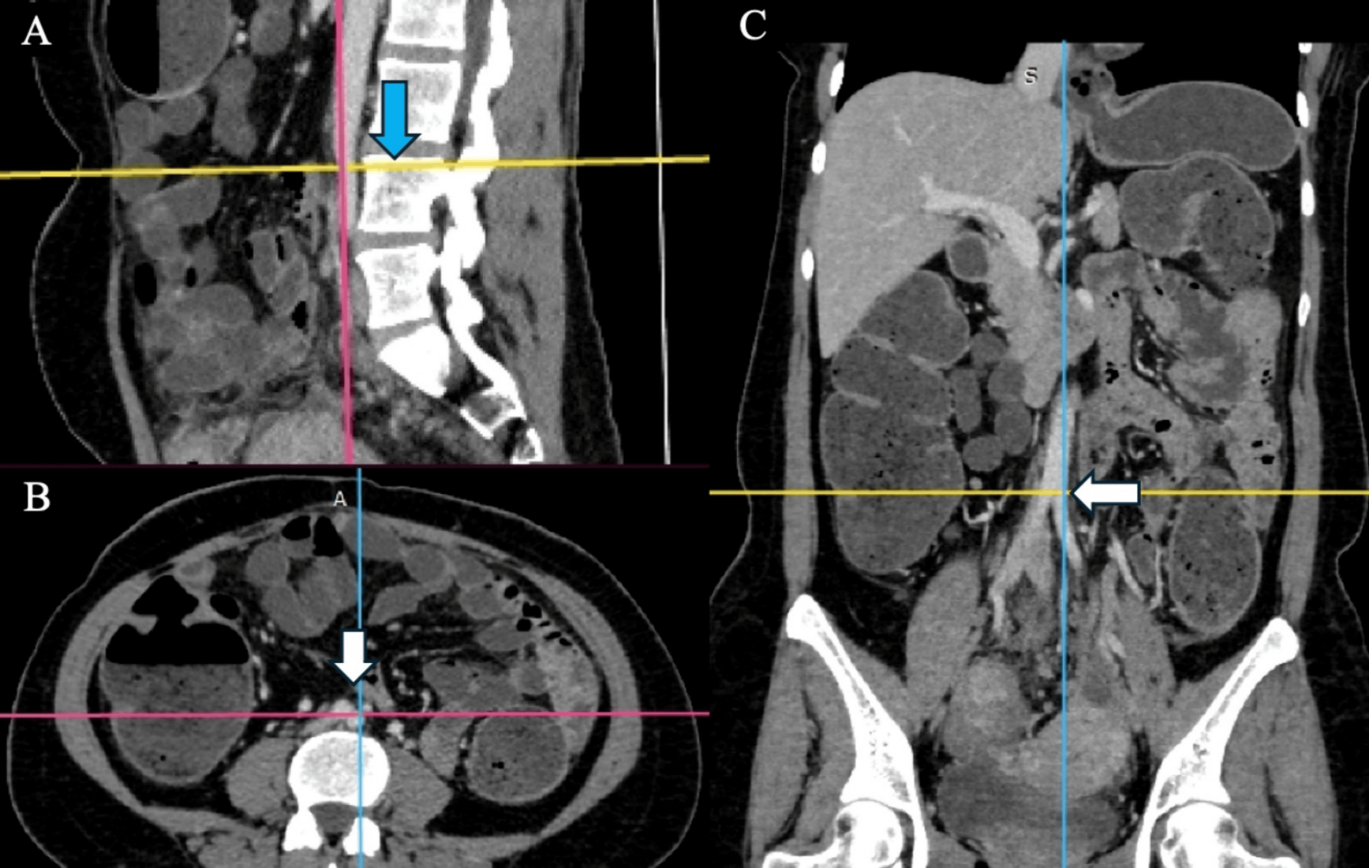
Typical AAB at the upper L4 vertebrae without AAD (A) AAB at the upper border of the L4 vertebrae in the sagittal plane (blue arrow). (B) AAB in the corresponding axial plane (white vertical arrow). (C) AAB with no AAD in the coronal plane (white horizontal arrow) AAB: abdominal aortic bifurcation; AAD: abdominal aortic deviation

**Figure 4:**
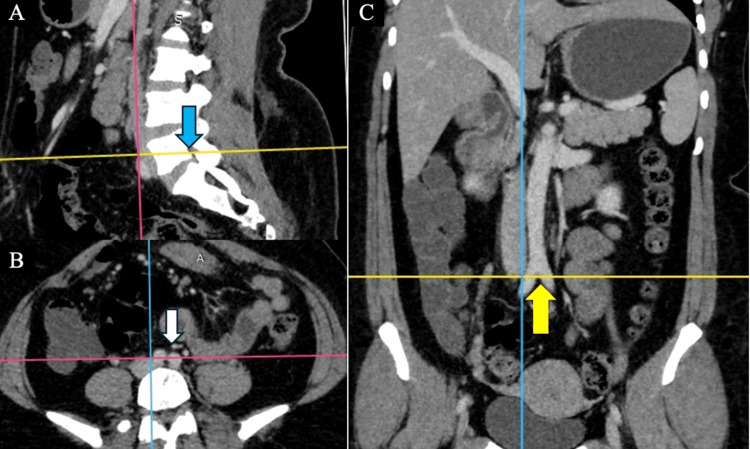
Low level of AAB at the middle of the L5 vertebrae in a subject without AAD (A) AAB at the middle of the L5 vertebrae in the sagittal plane (blue arrow). (B) AAB at the same corresponding level in the axial plane (white arrow). (C) AAB in the corresponding coronal section (yellow arrow) without AAD AAB: abdominal aortic bifurcation; AAD: abdominal aortic deviation

Figure 3 depicts a typical AAB at the upper border of the L4 vertebrae without AAD. Figure 4 depicts a low level of AAB, level with the middle of the L5 vertebrae.

## Discussion

The AA is the largest blood vessel in the abdomen. It begins at the aortic hiatus of the diaphragm, usually level with the 12th thoracic vertebrae, and descends anterior to the lumbar vertebra, most often bifurcating at the level of the fourth lumbar vertebrae into the right and left common iliac artery, slightly to the left of the midline. Several anatomical variations of the AA have been noted, such as differences in the angle of AAB, diameter of AA, deviation/tortuosity of AA, and variations in the level of AAB with respect to vertebral levels. Of these, variations in the level of AAB with respect to vertebral levels are of the most clinical significance [18].

Cadaveric studies have often been limited by the number of available cadavers and, additionally, are time- and resource-consuming. Often, cadaveric studies are not a true representation of the population in terms of age, sex, etc., as these factors often cannot be pre-determined and are therefore based on availability. Furthermore, changes in tissue architecture and preservation of cadavers hamper the interpretation of anatomical variations, and, since cadavers have usually been lying flat for a long time, the vertebral levels of bifurcation and other variations may not be truly representative of the observations that occur in live/surgical cases [12].

Advancements in three-dimensional cross-sectional imaging have made way for new avenues in the assessment and analysis of these vascular variations. While angiographic studies are more accurate in the demonstration of vascular variations, they are limited by access and complexities of technique. CECT scans of the abdomen are one of the most frequently undertaken radiological investigations and also give a reasonably clear picture of the vascular anatomy [4,5,17]. In this study, we used CECT scans of the abdomen in our cohort of patients in order to depict the anatomical variations of the AA and its branches. 

In our study cohort, we had almost equal representation of both sexes, 98 men (47%) versus 110 women (53%), and additionally had good representation of various age groups, with a median age of 50 years and a range of 19-78 years. The results of our study are generalizable to the normal population as well.

One of the most commonly studied variations of AA is the level of AAB with respect to vertebral levels. Notably, the level of AAB has been found to be profoundly affected by the presence/absence of AAD. As noted in the study by Ekingen and Çetinçakmak [5], the overall rate of AAD in the entire population was found to be 115 out of 721 subjects (16%), with a higher rate in women versus men (20.3% vs 14.37%; p=0.006). Although the authors did note a statistically significant difference in the level of AAB in men versus women in the cohort without AAD (p=0.001), the same was not observed in the cohort of cases with AAD (p=NS). There is a dearth of Indian studies evaluating the rate of AAD, and no previous study has reported relevant results. We evaluated the rate of aortic deviation in our cohort of patients and found the overall average to be 44 out of 208 (21.2%) for both sexes combined. In concordance with the results reported by Ekingen and Çetinçakmak [5], we also observed a higher rate of AAD among women (25.45%) compared to men (16.32%), but this was not statistically significant (p=0.108) unlike in Ekingen and Çetinçakmak's [5] study. We did note that the maximum frequency of AAD was observed in the 71-80-year age group (three out of seven subjects, 42.8%), compared with younger age groups, 11 out of 37 subjects (29.7%) for 31-40 years and 17 out of 61 (27.8%) for 51-60 years, with a statistical significance (p=0.0346). This is a novel finding, which has not been reported in any previous study on the Indian population. As individuals age, there is laxity of the musculature in the great vessels, which may contribute to the higher frequency of AAD in older individuals. 

AAB has traditionally been noted to be more frequent at the level of the L4 vertebrae. The majority of related studies have reported the level of bifurcation with respect to whole vertebral levels; in our study, we reported it more specifically, on different planes of the same vertebral levels (that is, the upper, middle, and lower levels of the respective vertebrae). We noted the most common level of AAB to be the middle of the L4 vertebrae, in 65 cases, AAB was located below the L4, and, in 36 cases (17.3%), it was above the L4 vertebrae. The highest level of AAB was observed at the L2-L3 intervertebral disc in one case (0.5%), and the lowest level was observed at the L5-S1 intervertebral disc in two cases (1%). Huang et al. and Degheili et al. reported the highest and lowest levels of AAB at the upper L2 vertebral body and S2 sacral vertebrae, respectively [7,8].

As in the majority of previous studies [4,5,9-12,19], we found the AAB level to be most frequently located at the L4 vertebra. Few studies have granularly reported the AAB level at L4. In a cadaveric study, Pirró et al. [19] reported AAB most frequently at the upper L4 in 21 cases (50%). In a CT angiographic study, Inamasu et al. [20] reported AAB at the lower L4 in 32 cases (32%). In an MRI-based study, Lee et al. [11] documented AAB at the upper half of L4 in 95 cases (45%). As illustrated in Table 6, we found AAB to be most commonly observed at the level of the middle L4 in 65 cases (31.2%), followed by the lower L4 in 49 cases (23.6%).

**Table 6:** Level of AAB with respect to vertebral levels in various studies AAB: abdominal aortic bifurcation; CT: computed tomography; MRI: magnetic resonance imaging; CECT: contrast-enhanced computed tomography

Study (year) (reference)	Type of study (country)	Number of cases	AAB level with respect to vertebral level	Remarks
Pirró et al. (2005) [19]	Cadaveric	42	Most frequent at the upper L4 (50%) followed by the upper L5 (39%)	-
Khamanarong et al. (2009) [9]	Cadaveric (Thailand)	187	At the L4 vertebral body in 70.12% of cases, 17.6% had AAB at the L5 vertebral body (the majority of these L5 cases were between the ages of 80 and 99 years)	132 men/55 women; average age 67.3 years (30-88 years)
Prakash et al. (2011) [21]	Cadaveric (India)	50	Most common aortic bifurcation was seen at L4 in 54%, L5 in 26%, and L3 in 20% subjects	-
Deswal et al. (2014) [12]	Cadaveric (India)	25	AAB at the L4 vertebrae in 64% of cases, L4-L5 IVD in 16% of cases, and L5 in 12% of cases	16 men/7 women
Inamasu et al. (2005) [20]	CT angiography	100	Most frequented level of AAB at the lower L4, upper L4, and L4-L5 IVD at 32%, 23%, and 23%, respectively	50 men and 50 women with age ranging from 20 to 88 years
Khader et al. (2022) [10]	CECT of the abdomen (Jordan)	100	The most common level of AAB is at L4 in 65% followed by L3-L4 IVD in 24% of cases. AAB is higher (at the L3 level) in women as compared to men (14% vs 8%)	50 men/50 women
Chithriki et al. (2002) [4]	MRI (USA)	441	AAB was observed at the L4 vertebral body in 67% of cases in this study. AAB was most commonly located at L4 (47%) followed by L4/L5 (33%) in cases with lumbarized sacral vertebrae (n=15). In cases with sacralized lumbar vertebrae (n=22), AAB was observed at L3 in 59% and at L4 in 23% of cases	Vertebrae counted from above (cervical) rather than below (L5/S1)
Lee et al. (2004) [11]	MRI	210	AAB at the upper half of L4 (45%), lower half of L4 (32%), L3-L4 IVD (10%), and L4-L5 IVD (4%)	Male-to-female ratio: 142:68
Ekingen and Çetinçakmak (2022) [5]	Angiography with multidetector CT (Turkey)	T21	AAB rate at the L3-L4 disc, upper L4, middle L4, lower L4, L4-5 disc, upper L5, and middle L5 was observed in 19.6%, 22.5%, 20.8%, 15%, 11.4%, 1.5%, and 0.5%, respectively	AAB was found to be at higher levels in women as compared to men (p=0.001)
Present study (2024)	CECT of the abdomen (India)	208	AAB is most commonly located in the middle of the L4 vertebrae (31.2%) followed by the L4 lower vertebrae (23.6%). In 59.1% of patients, AAB was located at the level of the L4 vertebrae (either upper, middle, or lower). In 23.6% of patients, the AAB was located at a level below the lower L4 vertebrae, and in 17.3% of patients, the AAB was located at a level above the upper L4 vertebrae	Male-to-female ratio: 98:110. Median age: 50 years (19-78 years)

Khader et al. [10] reported a higher level of AAB in women compared to men (14% vs 8%). Similarly, we noted that AAB was located at a higher vertebral level (above L4) in a greater proportion of women compared to men, although this finding was not statistically significant (p=0.346). In our study, no differences in the vertebral level of AAB were noted with respect to age (0.48) or AAD (p=0.55).

The retrospective nature of the study was one of the limitations, because it was not possible to collect other demographic data, such as height, weight, and BMI, which could have been valuable for the further analysis of variations and possibly led to more insights. Our research represents the first study to document the rate of AAD in the Indian population and its influence on AAB.

## Conclusions

In this retrospective, observational, single-center study conducted on 208 CECT scans of the abdomen and pelvis, we observed the rate of AAD to be 21.2% (women at 25.45% vs men at 16.32%) and the maximum AAD rate occurring in the 71-80-year age group. The most common level of AAB was noted at the level of the L4 vertebrae in approximately 60% of subjects. The results of our study could serve as a baseline for future research on AAD and AAB in Indian subjects.

## References

[1] Ozgüner G, Sulak O (2011). Development of the abdominal aorta and iliac arteries during the fetal period: a morphometric study. Surg Radiol Anat.

[2] Iasiello M, Vafai K, Andreozzi A, Bianco N (2017). Analysis of non-Newtonian effects within an aorta-iliac bifurcation region. J Biomech.

[3] Shah PM, Scarton HA, Tsapogas MJ (1978). Geometric anatomy of the aortic--common iliac bifurcation. J Anat.

[4] Chithriki M, Jaibaji M, Steele RD (2002). The anatomical relationship of the aortic bifurcation to the lumbar vertebrae: a MRI study. Surg Radiol Anat.

[5] Ekingen A, Çetinçakmak MG (2022). Level of variations of the aortic bifurcation and distance measurements between the aortic bifurcation and the common iliac bifurcations. J Anat Soc India.

[6] Arudchelvam J (2021). Study on the variations of the ventral abdominal aortic branches: a computed tomography based study. Sri Lanka J Surg.

[7] Degheili JA, Malhas H, Yoo TK, Dergham MY (2020). A very low-positioned aortic bifurcation. Vasc Specialist Int.

[8] Huang W, Ge G, Meng J, Xu Y (2010). High bifurcation of abdominal aorta upon horseshoe kidney at the level of upper L2 vertebral body: a rare case report. Surg Radiol Anat.

[9] Khamanarong K, Sae-Jung S, Supa-Adirek C, Teerakul S, Prachaney P (2009). Aortic bifurcation: a cadaveric study of its relationship to the spine. J Med Assoc Thai.

[10] Khader M, Al-Hyasat TG, Salameh IY (2022). Variations in the bifurcation level of the abdominal aorta, formation level of the inferior vena cava, and insertion level of the left renal vein into the inferior vena cava and their clinical importance in laparoscopic surgery. Laparoscopic, Endoscopic and Robotic Surgery.

[11] Lee CH, Seo BK, Choi YC (2004). Using MRI to evaluate anatomic significance of aortic bifurcation, right renal artery, and conus medullaris when locating lumbar vertebral segments. AJR Am J Roentgenol.

[12] Deswal A, Tamang BK, Bala A (2014). Study of aortic- common iliac bifurcation and its clinical significance. J Clin Diagn Res.

[13] Shakeri A, Shakeri M, Ojaghzadeh Behrooz M, Behzadmehr R, Ostadi Z, Fouladi DF (2018). Infrarenal aortic diameter, aortoiliac bifurcation level and lumbar disc degenerative changes: a cross-sectional MR study. Eur Spine J.

[14] Forbang NI, McClelland RL, Remigio-Baker RA (2016). Associations of cardiovascular disease risk factors with abdominal aortic calcium volume and density: the Multi-Ethnic Study of Atherosclerosis (MESA). Atherosclerosis.

[15] Sharafuddin MJ, Hoballah JJ, Kresowik TF, Sharp WJ, Golzarian J, Sun S, Corson JD (2008). Long-term outcome following stent reconstruction of the aortic bifurcation and the role of geometric determinants. Ann Vasc Surg.

[16] Taylor A, Rockall AG, Powell ME (2007). An atlas of the pelvic lymph node regions to aid radiotherapy target volume definition. Clin Oncol (R Coll Radiol).

[17] Joon P, Rastogi R, Pratap V, Gupta Y, Wani AM (2017). Multidetector computed tomography (MDCT) evaluation of anomalies of abdominal aorta & its major branches. Ann Int Med Den Res.

[18] Kornafel O, Baran B, Pawlikowska I, Laszczyński P, Guziński M, Sąsiadek M (2010). Analysis of anatomical variations of the main arteries branching from the abdominal aorta, with 64-detector computed tomography. Pol J Radiol.

[19] Pirró N, Ciampi D, Champsaur P, Di Marino V (2005). The anatomical relationship of the iliocava junction to the lumbosacral spine and the aortic bifurcation. Surg Radiol Anat.

[20] Inamasu J, Kim DH, Logan L (2005). Three-dimensional computed tomographic anatomy of the abdominal great vessels pertinent to L4-L5 anterior lumbar interbody fusion. Minim Invasive Neurosurg.

[21] Prakash Prakash, Mokhasi V, Rajini T, Shashirekha M (2011). The abdominal aorta and its branches: anatomical variations and clinical implications. Folia Morphol (Warsz).

